# Beckwith-Wiedemann syndrome and isolated hemihyperplasia

**DOI:** 10.1590/S1516-31802003000300010

**Published:** 2003-05-01

**Authors:** Marcus Vinícius de Matos Gomes, Ester Silveira Ramos

**Keywords:** Beckwith-Wiedemann syndrome, Imprinting, Methylation, Isolated hemihyperplasia, Cancer, Síndrome de Beckwith-Wiedemann, Imprinting, Metilação, Hemihiperplasia isolada, Câncer

## Abstract

**CONTEXT:**

Beckwith-Wiedemann syndrome is a complex and heterogeneous overgrowth syndrome with genetic and epigenetic alterations, involving genomic imprinting and cancer predisposition. Isolated hemihyperplasia is of unknown cause, and it may represent a partial or incomplete expression of Beckwith-Wiedemann syndrome.

**OBJECTIVES:**

A clinical and molecular review and proposal of the use of an experimental protocol to provide a practical approach for the physician.

**DATA SYNTHESIS:**

This review demonstrates the genetic and epigenetic mechanisms involved in the Beckwith-Wiedemann syndrome and isolated hemihyperplasia, and the candidate genes. To our knowledge, this is the first Brazilian protocol for research into these disorders. The results have been used at the Faculdade de Medicina de Ribeir ão Preto, Universidade de São Paulo, to elucidate the basis of Beckwith-Wiedemann syndrome and isolated hemihyperplasia, and have been applied at the Hospital Universitário of the Faculdade de Medicina.

**CONCLUSIONS:**

Elucidation of the etiological mechanisms and use of a laboratory protocol to detect alterations in these disorders may be useful for guiding the management of such patients and genetic counseling of the families.

## RESUMO

**CONTEXTO:**

A síndrome de Beckwith-Wiedemann é uma doença de hipercrescimento complexa e heterogênea com alterações genéticas e epigenéticas, envolvendo *imprinting* genômico e predisposição ao câncer. A causa da hemi-hiperplasia isolada ainda é desconhecida e pode representar uma expressão parcial ou incompleta da síndrome de Beckwith-Wiedemann.

**OBJETIVOS:**

Revisão clínica e molecular e proposta de um protocolo experimental para utilização na prática médica.

**SÍNTESE DOS DADOS:**

Esta revisão apresenta os mecanismos genéticos e epigenéticos envolvidos na síndrome de Beckwith-Wiedemann e na hemi-hiperplasia isolada, os genes candidatos e o primeiro protocolo brasileiro, de que temos conhecimento, para a pesquisa dessas doenças. Os resultados têm sido utilizados na Faculdade de Medicina de Ribeirão Preto da Universidade de São Paulo para elucidar as bases da síndrome de Beckwith-Wiedemann/hemi-hiperplasia isolada e aplicados no Hospital das Clínicas desta Faculdade.

**CONCLUSÕES:**

A elucidação dos mecanismos etiológicos e o uso de um protocolo laboratorial para se detectar as alterações nessas doenças pode ser proveitoso no acompanhamento clínico dos pacientes e no aconselhamento genético das famílias.

## INTRODUCTION

### Beckwith-Wiedemann syndrome

Beckwith-Wiedemann syndrome is a congenital disorder first recognized in 1964 by Dr. H.R. Wiedemann,^1^ a geneticist, and then in 1969 by Dr. J. Bruce Beckwith,^2^ a pediatric pathologist. The syndrome is usually sporadic, but may be inherited.^3^ The incidence has been report to be approximately 1:13,700 births.^4^

The most common phenotypes associated are:

- Macroglossia: larger than usual for an infant, may disturb the child's ability to eat, breathe or speak. In severe cases, corrective surgery may be necessary. The enlargement is no longer apparent by 6-8 years of age.
- Abdominal wall defects (omphalocele, umbilical hernia, diastasis recti).
- Increased growth: birth weight and length (usually above the mean), visceromegaly (particularly kidneys, liver, and pancreas) and hemihyperplasia (usually all or part of one side of the body).
- Ear lobe creases, first described by Irving (1967) and then used as one of the criteria for diagnosis of the illness, and/or pit indentations on the posterior helix.^5-8^

**Figure 1:**
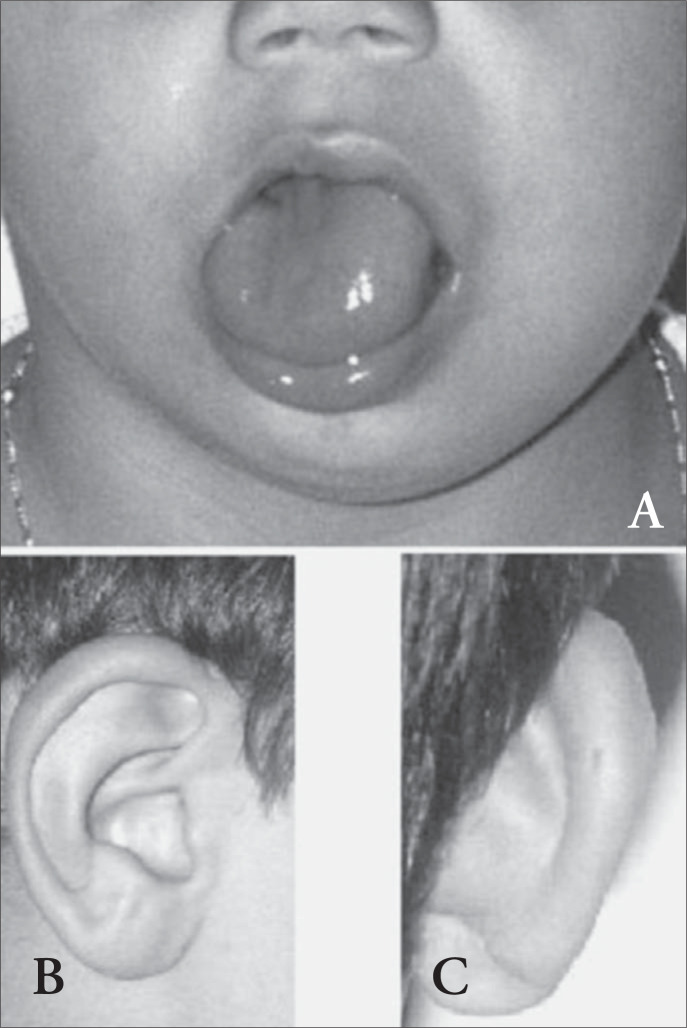
Common phenotypes associated with Beckwith-Wiedemann syndrome: A) macroglossia and facial/tongue hemihyperplasia, B) ear lobe creases, C) pit on posterior helix.

Macroglossia, together with omphalocele or other umbilical abnormalities, allows recognition of the disorder at birth. Sometimes hemihyperplasia is not present at birth but becomes apparent later in childhood. It can be noted in approximately 12.5% of the cases and is present in 40% of the children who develop tumors (Figure 1).^9^

The risk situations are:

- Hypoglycemia, which occurs shortly after birth. Although it is usually temporary, if untreated, it can cause seizures and hypoxia, leading to severe complications, including mental deficiency.^10^
- Premature birth and its complications.
- Development of tumors: children with Beckwith-Wiedemann syndrome have a 7-21% risk for the development of embryonic malignancies, most notably Wilms’ umor of the kidney,^11-13^ although a wide variety of benign and malignant tumors have been reported, including hepatoblastoma, adrenocortical carcinoma, rhabdomyosarcoma and neuroblastoma.^8,13^

### Isolated hemihyperplasia

Hemihyperplasia is clinically defined as asymmetric overgrowth of one or more body parts. The overgrowth may involve an entire half of the body, a single limb, and/or one side of the face or combinations thereof. There may be accompanying asymmetric visceromegaly.^14^

It may be an isolated finding, or it may be associated with a variety of multiple malformation syndromes. Rowe et al (1962)^15^ proposed a classification system for hemihyperplasia, based on anatomic site of involvement: a) complex hemihyperplasia – involvement of half of the body (at least one arm and one leg), in which the affected parts may be contra or ipsilateral; b) simple hemihyperplasia – involvement of a single limb; c) hemifacial hyperplasia – involvement of one side of the face. The incidence of isolated hemihyperplasia has been report to be approximately 1:86,000 live births^16,17^ and increased birth weight has been observed (mean of 3.8 kg).^18^ Hoyme et al (1998)^17^ reported an increase in the incidence of certain abdominal tumors in children with isolated hemihyperplasia (5.9%), in comparison with the general population.

### Genetic and epigenetic aspects

Beckwith-Wiedemann syndrome is a disorder with complex genetic and epigenetic alterations that occur in the chromosomal region 11p15.5. Genomic imprinting is a phenomenon that causes genes to be expressed according to their parental origin. Approximately 40 imprinted genes have now been identified in the human genome, with roles in prenatal growth, development of particular cell lineages, and in human diseases.^19^ The imprinting mechanism is still unclear, but probably involves differential methylation of specific sites in or near imprinted genes. It is characterized by a methylation pattern specific for the parental allele. Uniparental disomy is another phenomenon that involves and displays imprinting effects. It occurs when a pair of homologous chromosomes is inherited from the same parent. There are several mechanisms that could give rise to uniparental disomy, but the most common is “trisomic rescue”, involving the loss of a supernumerary chromosome from a trisomic conceptus, leaving two homologues from the same gamete.^20^

**Figure 2:**
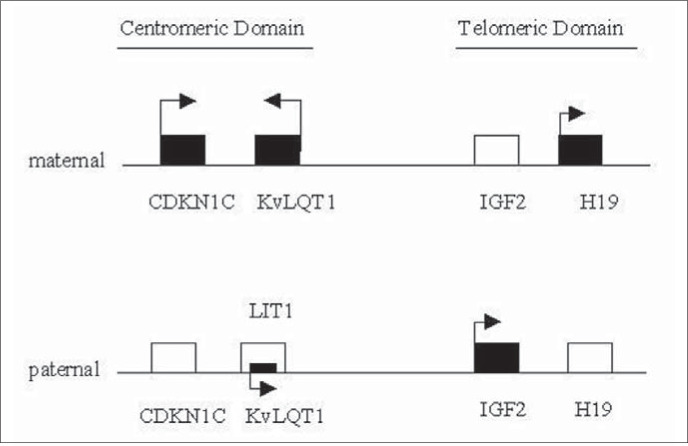
Telomeric and centromeric domains involved in Beckwith-Wiedemann syndrome. Expressed genes are indicated by open boxes, and silenced genes by closed ones. The arrows indicate the transcriptional orientation.

The chromosomal region 11p15.5 is divided into two imprinted gene clusters: IGF2 and H19 are mapped in the telomeric domain; and CDKN1C (also know as p57^kip2^), KVLQT1 and LIT1 (also know as KvLQT 1AS) in the centromeric domain (Figure 2) (Table 1).^21-32^ Genes IGF2 and H19 are coordinately regulated in normal tissues through an expression competition model.^33,34^ On the expressed maternal H19 allele, the differential methylated region (H19DMR), mapped 2 kb upstream from this gene, is nonmethylated and is able to bind to an insulator protein CCCTC-binding factor termed CTCF. This insulates the maternal IGF2 promoter from enhancers located 3’ from the H19 gene, and prevents expression of IGF2. On the paternal allele, the H19DMR is methylated. This blocks the binding of the CTCF protein and prevents expression of the imprinted paternal H19 alleles.^35^ The IGF2 promoter now has access to the 3’ enhancers and the paternal allele is expressed via enhancers and boundary elements.^36^ This suggests that H 19DMR may function as a regional imprinting center that regulates the telomeric 11p15 imprinted domain. Deletion of the entire DMR results in loss of imprinting, with activation of H19 expression and reduction of IGF 2 paternal expression when paternally inherited. The IGF2 maternal allele is activated and the maternal H19 expression is reducted when the deletion is maternally inherited.^37,38^ In the centromeric domain, the KvDMR1, a maternally methylated CpG island at the 5’ end of the LIT1 transcript, may function as a regional imprinting center. Loss of methylation of KvDMR1 has been associated with biallelic expression of LIT1 in some Beckwith-Wiedemann syndrome cases.^31^

**Table 1:** Candidate genes in Beckwith-Wiedemann syndrome and in isolated hemihyperplasia

Gene	Map	Expression	Product	Characteristics
IGF2	11p15.5 - tel	pat	Insulin-like growth factor II	Hyperexpression in 50% of sporadic cases of Beckwith-Wiedemann syndrome^24,25,26^ and in several tumors.^27,28^
H19	11p15.5 - tel	mat	Nontranslated mRNA	Tumor suppressor gene.^29^ Harbors an IC region.^37^
CDKN1C	11p15.5-cent	mat	Cyclin-dependent kinase inhibitor	Cell cycle regulator.^30^
KvLQT1	11p15.5-cent	mat	Potassium channel	Harbors an IC region.^24^
LIT1	11p15.5-cent	pat	Antisense transcript	Lies within the KvlQT1 gene.^31,32^

*Note: IC = imprinting control; tel = telomeric; cent = centromeric; pat = paternal; mat = maternal.*

### Etiology of Beckwith-Wiedemann syndrome and isolated hemihyperplasia

The etiology of Beckwith-Wiedemann syndrome is complex and genetic and/or epi-genetic alterations have been described. In approximately 15% of patients, Beckwith-Wiedemann syndrome is inherited as an autosomal dominant pattern with variable expressivity and incomplete penetrance; 2% of patients have cytogenetics abnormalities and the remainder are sporadic. Imprinted genes have been implicated in the pathogenesis of both familial and sporadic Beckwith-Wiedemann syndrome (Table 2).^39-47^ Isolated hemihyperplasia and other mild manifestations of Beckwith-Wiedemann syndrome may represent patchy overexpression of the IGF2 gene following defective imprinting.^17^ The frequency of hemihyperplasia among Beckwith-Wiedemann syndrome patients with unipa-rental disomy or aberrant methylation of both H 19 and LIT1 has been found to be significantly higher than that in patients without such defects.^48^ Defects in imprinting are also related to the development of a number of cancers, including Wilms’ tumor, neuroblastoma, acute myeloblastic leukemia, and rhabdomyosarcoma.^49,50^ Many authors have hypothesized that there may be a relationship between cancer risk and loss of imprinting of IGF 2 and/or aberrant methylation of the H 19DMR, because both defects have been described in cancers detected in children with Beckwith-Wiedemann syndrome, especially Wilms ’ tumor.^39,51-53^ Imprinting defects in the centromeric domain (KvDMR) of Beckwith-Wiedemann syndrome patients have been observed in rhabdomyosarcoma, hepatoblastoma and gonadoblastoma, but not in Wilms’ tumor. This suggests that distinct tumor predisposition profiles result from deregulation of the telomeric domain versus the centromeric domain and that these imprinting defects activate distinct genetic pathways for embryonic tumorigenesis.^13^

**Table 2:** Mechanisms involved in Beckwith-Wiedemann syndrome (alterations and results)

Alteration	Results
Abnormal methylation of H19DMR.^39,40^	Allows access by the IGF2 maternal allele to the enhancer.^41,42^
Loss of imprinting of IGF2	Activation of the IGF2 maternal allele.^25,28,31^ Hyperexpression of IGF2 in mice results in the spectrum of the Beckwith-Wiedemann syndrome phenotype.^43^
Chromosomal rearrangements	Disruption of imprinted gene expression.^44^
CDKN1C mutations	Present in familial cases of Beckwith-Wiedemann syndrome.^45,46^
Loss of imprinting of LIT1	Linked to abnormal methylation of KvDMR.^31^
Uniparental disomy of 11p15	Gene hyperexpression by activation of a normally silenced allele.^47^

*Note: DMR = differential methylated region.*

## METHOD

After analyzing the papers in the literature, we created an experimental protocol for the identification of Beckwith-Wiedemann syndrome and isolated hemihyperplasia, and to elucidate the mechanisms involved in the condition. This protocol has been used at the Department of Genetics of the Faculdade de Medicina de Ribeirão Preto, Universidade de S ão Paulo, and for the management of the patients and genetic family counseling in the Genetic Outpatient Service of the Hospital Universit ário of the Faculdade de Medicina de Ribeirão Preto, Universidade de São Paulo.

## PATIENTS

For those families who have agreed to participate in the protocol, an informed consent and medical-information release document has been obtained, as approved by the respective institutional review boards. For the purpose of such studies, we define patients as having Beckwith-Wiedemann syndrome if a clinical diagnosis has been made by a physician. The following specific criteria also need to be fulfilled: presence of 3 major findings (macroglossia, pre or postnatal growth > 90^th^ percentile, and abdominal wall defects) or 2 major features plus 3 or more minor features such as ear signs (ear lobe creases or posterior helical ear pits), facial nevus flammeus, hypoglycemia, nephromegaly and hemihyperplasia .^6,56,55^ Patients with isolated hemihyperplasia or asymmetric overgrowth have also been analyzed, because they share a predisposition with Beckwith-Wiedemann syndrome patients for the same neoplasia, especially Wilms’ tumor. The mechanism involved in isolated hemihyperplasia may have links with the mechanism for Beckwith-Wiedemann syndrome.

## CLINICAL MANAGEMENT

Prenatal ultrasound has occasionally been helpful when certain characteristics (omphalocele, enlarged abdominal circumference, enlarged kidneys, protruding tongue) are present. Monitoring the glycemia in Beckwith-Wiedemann syndrome newborns every 6 hours during the first 3 days, in order to correct blood glucose levels, can prevent complications in the central nervous system.^10^ Everman et al (2000)^56^ conducted a study of serial alpha-fetoprotein concentration in 22 Beckwith-Wiedemann patients. The concentration was greater in Beckwith-Wiedemann syndrome patients and declined during the postnatal period at a significantly slower rate than in healthy children. Tongue correction surgery may be necessary in some severe cases. Follow-up by ultrasound is warranted in all patients with Beckwith-Wiedemann syndrome, especially in cases associated with hemihyperplasia and/or nephromegaly, and in patients with isolated hemihyperplasia due to predisposition to embryonic tumors, including Wilms’ tumor. The screened patients should be submitted to at least 2 ultrasounds per year up to the age of 5-7 years, and then annually until puberty. Some screening sonogram programs use intervals of 4 months or less.^57-59^

### Cytogenetic analysis

Conventional cytogenetic analysis of peripheral blood lymphocytes is performed^60^ and the slides are subjected to standard Giemsa staining .^61^ High resolution banding and in-situ fluorescence hybridization may be used in specific cases. The karyotype description should follow the nomenclature recommended by the International System for Human Cytogenetic Nomenclature (1995).

### Molecular analysis

**Figure 3:**
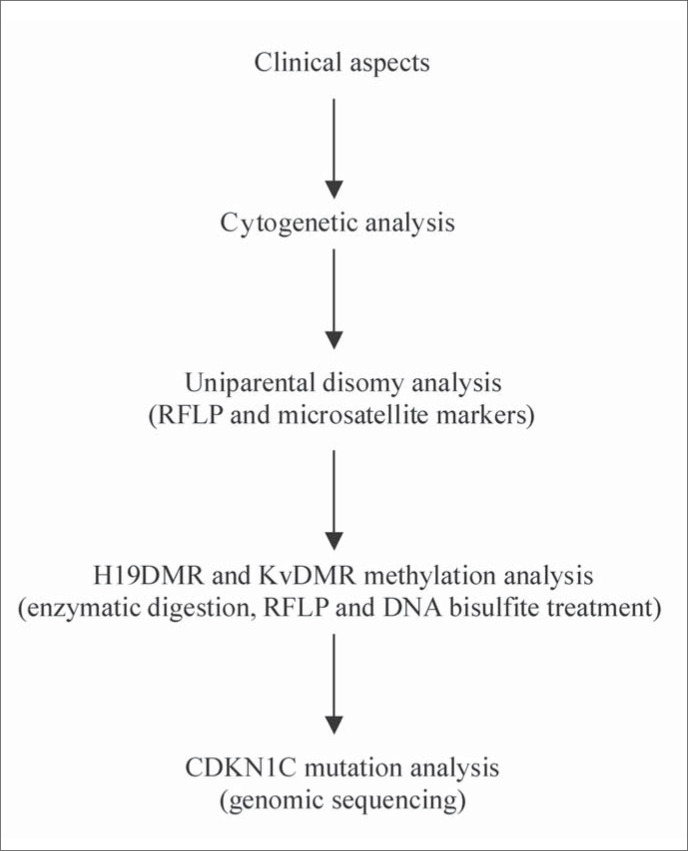
Protocol for Beckwith-Wiedemann syndrome analysis.

Genomic DNA is isolated from peripheral-blood lymphocytes. In the analysis of uniparental disomy, patient, paternal and maternal samples are genotyped by *ApaI*/IGF2 and *RsaI*/H19 restriction fragment length polymorphism. Non-informative cases are genotyped with at least two microsatellite markers. IGF2 gene expression analysis (skin fibroblast RNA extraction) allows the detection of loss of imprinting. This is a highly invasive methodology that requires biopsy. For this reason we use it only in specific cases. As an alternative to this, we can use less invasive methodology based on the relationship between IGF2 and H19 correlated expression. Digestion using methylation-sensitive and nonsensitive enzymes and bisulfite DNA treatment allows the analysis of H19DMR and KvDMR methylation, and gene sequencing allows the analysis of CDKN1C mutations (Figure 3).

## DISCUSSION/CONCLUSION

Beckwith-Wiedemann syndrome is an overgrowth syndrome with tumor predisposition in childhood. It can be inherited but most cases are sporadic. The etiology is not totally clear, but some genetic and epi-genetic aspects may be involved, like chromosomal rearrangements in the 11p15.5 region (2%), mutations and imprinting defects of genes, most probably involving methylation of the differential methylated region and uniparental disomy. Isolated hemihyperplasia is of unknown cause, and it may represent a partial or incomplete expression of Beckwith-Wiedemann syndrome. The heterogeneous genetic and epi-genetic basis of Beckwith-Wiedemann syndrome and isolated hemihyperplasia hampers understanding of the mechanisms involved in these disorders. Knowledge of the clinical and molecular basis of Beckwith-Wiedemann syndrome and isolated hemihyperplasia, establishment of a relationship between the causes of Beckwith-Wiedemann syndrome and etiology of isolated hemihyperplasia, with an experimental protocol for their study, may be useful for patient management and better genetic counseling of their families.
